# Extract of *Isatidis Radix* Inhibits Lipid Accumulation in In Vitro and In Vivo by Regulating Oxidative Stress

**DOI:** 10.3390/antiox12071426

**Published:** 2023-07-14

**Authors:** Yo-Han Han, Ji-Ye Kee

**Affiliations:** 1 Department of Pharmacology, College of Korean Medicine, Kyung Hee University, Seoul 02447, Republic of Korea; yhan@khu.ac.kr; 2 Department of Oriental Pharmacy, College of Pharmacy, Wonkwang-Oriental Medicines Research Institute, Wonkwang University, Iksan 54538, Republic of Korea

**Keywords:** *Isatidis Radix*, antioxidant, ROS, AMPK, human adipose mesenchymal stem cells

## Abstract

*Isatidis Radix* (IR), the root of *Isatis tinctoria* L. belonging to Brassicaceae, has been traditionally used as a fever reducer. Although some pharmacological effects, such as anti-diabetes, anti-virus, and anti-inflammatory, have been reported, there is no study on the anti-obesity effect of IR. This study used 3T3-L1 cells, human mesenchymal adipose stem cells (hAMSCs), and a high-fat diet (HFD)-induced obese mouse model to confirm the anti-adipogenic effect of IR. Intracellular lipid accumulation in 3T3-L1 cells and hAMSCs was decreased by IR treatment.IR extract especially suppressed reactive oxygen species (ROS) production through a cluster of differentiation 36 (CD36)-AMP-activated protein kinase (AMPK) pathway. Consequently, the expressions of peroxisome proliferator-activated receptor gamma (PPARγ), CCAAT-enhancer-binding proteins alpha (C/EBPα), and fatty acid synthesis (FAS) were inhibited by IR extract. In addition, β-oxidation-related genes were also decreased by treatment of IR extract. IR inhibited weight gain through this cascade in the HFD-induced obese mouse model. IR significantly suppressed lipid accumulation in epididymal white adipose tissue (eWAT). Furthermore, the administration of IR extract decreased serum free fatty acid (FFA), total cholesterol (TC), and LDL cholesterol, suggesting that it could be a potential drug for obesity by inhibiting lipid accumulation.

## 1. Introduction

Although weight gain can be prevented in advance, nearly one-third of the world’s people still suffer from overweight and obesity [1]. Obesity is characterized by the accumulation of lipids in cells. Therefore, it is important to understand the mechanisms of adipocyte differentiation and intracellular lipid accumulation to treat and prevent obesity [2]. Obesity is recognized as a major cause of various metabolic diseases, such as type 2 diabetes, hypertension, and cardiovascular disease, and is recognized as a critical disease in itself [3]. Several reports also indicated that obesity could cause various cancers, such as colorectal, breast, prostate, and kidney cancer. In addition, obesity can make cancer even worse [4]. Moreover, due to appearance problems, psychological disorders, such as depression, anthropophobia, and sleep disorders, can be induced by obesity [5]. Therefore, it is important to prevent obesity for physical and psychological health.

Increased oxidative stress and reactive oxygen species (ROS) can trigger the deposition of white adipose tissue (WAT) in both in vitro and in vivo studies, demonstrating that oxidative stress could induce adipocyte differentiation as well as enlargement of mature adipocyte size [6,7]. ROS production has been reported to activate the expression of CCAAT-enhancer-binding proteins beta (C/EBPβ), which is required to initiate early adipocyte differentiation [8]. A cluster of differentiation (CD) 36 is a membrane receptor protein highly expressed in various cells, such as adipocytes, hepatocytes, and some epithelia. CD36 participates in physiological mechanisms, including lipid metabolism, atherosclerosis, and angiogenesis [9]. CD36 mediated uptake of cellular free fatty acids (FFAs) and increased intracellular FFA-produced cytosolic and mitochondrial ROS [10]. In addition, the absence of CD36 significantly suppressed the adipogenic transcription factors, such as PPARγ and CCAAT-enhancer-binding proteins alpha (C/EBPα) [11,12]. Peroxisome proliferator-activated receptor gamma (PPARγ) cooperated with C/EBPα to induce adipocyte differentiation by increasing each other’s expressions [13,14,15]. Subsequently, these two factors, PPARγ and C/EBPα, induce expressions of downstream genes, such as fatty acid synthesis (FAS) [16].

AMP-activated protein kinase (AMPK) is important for maintaining energy homeostasis by promoting the consumption of ATP [17]. AMPK is activated by AMP/ATP ratio changes and upstream kinase, liver kinase B1 (LKB1) [18]. AMPK involves cellular antioxidant pathways to prevent inflammation pathway. AMPK is especially closely related to various metabolic phenomena in the body [19,20]. AMPK inhibits the adipogenic differentiation of pre-adipocytes by suppressing the expressions of PPARγ and C/EBPα [21]. For these reasons, activating AMPK is an important therapeutic target for treating obesity.

*Isatidis Radix* (IR), a root of the *Isatis tinctoria* L. or *Isatis indigotica* Fort, has been used as traditional herbal medicine. It has cured headaches, fever, and sore throats in East Asia [22]. Recently, some pharmaceutical effects were reported that IR exerted antioxidant, anti-virus, and anti-inflammatory effects [23,24]. In addition, Li et al. reported that polysaccharides extracted from IR alleviated type 2 diabetes mellitus by improving insulin resistance [25]. Even though various biological effects of IR have been studied, there was no study on lipid metabolism regarding oxidative stress and weight-reducing effect. In this study, we used 3T3-L1 cells, human adipose tissue-derived mesenchymal stem cells (hAMSCs), and high fat diet (HFD)-induced obese mice to confirm whether IR could inhibit weight gain by regulating lipid metabolism and oxidative stress.

## 2. Materials and Methods

### 2.1. Chemical Reagents

Oil-Red O solution, 3-isobutyl-1-methylxanthine (IBMX), insulin, dexamethasone (Dex), indomethacin, 3,3′,5-Triiodothyronine (T3), and troglitazone (Trog) were bought from Sigma Chemicals Co. (St. Louis, MO, USA). Epigallocatechin-3-gallate (EGCG) was purchased from Chengdu Biopurify Phytochemicals Ltd. (Chengdu, China). Bovine serum (BS), fetal bovine serum (FBS), Dulbecco’s modified Eagle’s medium (DMEM), and penicillin-streptomycin were purchased from Gibco BRL (Grand Island, NY, USA). Anti-GAPDH antibodies were purchased from Santa Cruz Biotechnology (Santa Cruz, CA, USA). Antibodies for pAMPK, pLKB1, PPARγ, and C/EBPα were purchased from Cell Signaling Technology (Beverly, MA, USA).

### 2.2. Preparation of IR

Dried IR was purchased from Omniherb (Uiseong, Republic of Korea). To prepare the water extract of IR (WIR), 100 g of IR was extracted with 1 L of water by heating at 100 °C for 3 h. The extract was frozen for drying, and the resulting powder was used as the total crude extract. The powder was dissolved in distilled water for treatment. To prepare the ethanol extract of IR (EtIR), 100 g of IR was extracted with 70% ethanol for 3 h and evaporated under reduced pressure. The remaining solution was frozen for drying. The resulting powder was dissolved in 20% DMSO at a high concentration and then diluted to experimental concentration using PBS. During the experiment, the final volume of DMSO did not exceed 0.05%. The yield was 18.6% for WIR and 27.12% for EtIR.

### 2.3. Differentiation of 3T3-L1 Cells and hAMSCs

The 3T3-L1 pre-adipocytes were obtained from American Type Culture Collection (Rockville, MD, USA). Cells were cultured in 10% BS/DMEM at 37 °C in a 5% CO_2_ atmosphere. Pre-adipocytes were seeded and differentiated as we previously described [26]. Briefly, 3T3-L1 pre-adipocytes were cultured until confluence. Two days after confluence (day 0), the cells were differentiated by a 10% FBS/DMEM containing MDI mixture consisting of 0.5 mM IBMX, 1 μM Dex, and 1 μg/mL insulin. After two days, the media were changed to 10% FBS/DMEM containing 1 μg/mL insulin every 2 days until day 6. WIR and EtIR were treated from day 2 to day 6 and then used for further analysis. hAMSCs were purchased from CEFO Bio (Seoul, Republic of Korea) and differentiated as previously described [16]. Briefly, the cells were cultured using hAMSCs growth media (CEFO Bio, Seoul, Republic of Korea) until confluence in 6-well plates (day 0). Then, the cells were differentiated using a differentiation media (DM) (10% FBS/DMEM, 0.5 mM IBMX, 1 μM Dex, 1 μM insulin, and 100 μM indomethacin) for 6 days. After six days, the cells were treated with WIR or EtIR in 1 μg/mL of insulin-containing 10% FBS/DMEM. This insulin-containing media was replaced every 2 days with WIR and EtIR until day 14.

### 2.4. Cell Viability 

Cell viability was confirmed by Cell Proliferation MTS Kit (Promega Corporation, Madison, WI, USA). We seeded 3T3-L1 cells and hAMSCs into a 96-well plate (5 × 10^3^ cells/well) and maintained in 10% FBS/DMEM for 24 h. Then, the cells were treated with various concentrations of WIR and EtIR, and media was replaced every 2 days with WIR and EtIR. After 4 days (3T3-L1 cells) and 8 days (hAMSCs), periods which are comparable to the anti-obesity effects test groups, 10 μL MTS was treated in each well for 4 h. The formazan concentration was measured at 490 nm by a VERSA max microplate reader (Molecular Devices, Sunnyvale, CA, USA).

### 2.5. Oil Red O Staining

To measure the lipid amount in 3T3-L1 cells and hAMSCs after differentiation fully, the Oil Red O staining method was conducted. Cells were fixed using 10% formaldehyde for 2 h and then were washed using 60% isopropanol. After staining with 0.3% Oil Red O solution for 30 min at room temperature, the cells were washed with distilled water. Stained cells were observed by a microscope (Leica, Wetzlar, Germany). After taking microscopic images, stained Oil Red O solution was extracted with 100% isopropanol (2 mL/well). A VERSA max microplate reader calculated the extracted Oil Red O solution at 500 nm.

### 2.6. Measurement of Cellular ROS Production

Cellular ROS was measured using the CellROX™ Green Reagent (Invitrogen, Waltham, MA, USA) through flow cytometric analysis according to the manufacturer’s instructions. Cells were treated with 5 μM of CellROX^®^ Reagent, and the cells were incubated for 30 min at 37 °C. Then, the media was removed and washed 3 times with PBS. Cells were collected and analyzed using Cytoflex Flow Cytometer (Beckman Coulter, Brea, CA, USA).

### 2.7. ROS Assay

The production of intracellular ROS was confirmed by a fluorometric intracellular ROS kit (Sigma-Aldrich, St. Louis, MO, USA, product number MAK143) according to the manufacturer’s instructions. After finishing the treatment of WIR and EtIR, the master reaction mix was added, and cells were incubated for 2 h at a 37 °C CO_2_ incubator. After incubation, the fluorescence intensity was measured on a SpectraMax Spectrofluorometer (Molecular Devices, Sunnyvale, CA, USA) with an excitation wavelength of 490 nm and an emission wavelength of 525 nm.

### 2.8. RNA Extraction and Real-Time RT PCR

Total RNA was isolated using a QIAzol lysis reagent (QIAGEN Sciences, Germantown, MD, USA). RNA was synthesized to first-strand cDNA using a Power cDNA Synthesis Kit (Applied Biosystems, Foster City, CA, USA) according to the manufacturer’s instructions. Genes were confirmed with a StepOnePlus Real-time RT-PCR System (Applied Biosystems, Foster City, CA, USA), and the relative expression levels were calculated by the ddCt method. GAPDH and h36b4 were used as endogenous control. The sequence of primers is indicated in Table 1.

### 2.9. Western Blot Analysis

Proteins were extracted using a protein extraction solution (iNtRon Biotech, Seoul, Republic of Korea) according to the manufacturer’s instructions. After adjusting to the same concentration, the protein solutions were mixed with SDS-PAGE protein-loading buffer and boiled at 95 °C for 5 min. The proteins were separated in an SDS-polyacrylamide gel and transferred to a PVDF membrane (Millipore, Billerica, MA, USA). To confirm various target proteins at the same time, the membranes were cut into several parts based on target protein size and protein marker. Using 5% BSA/TBS plus tween 20 (TBST), membranes were blocked and reacted with primary antibodies overnight at 4 °C. After washing using 0.1% TBST, membranes were reacted with appropriate secondary antibodies (horseradish peroxidase-conjugated anti-rabbit or anti-mouse immunoglobulin G (Dako, Glostrup, Denmark). After developing the membranes using an ECL solution (Santa Cruz, CA, USA), images were captured by the FluorChem E system (ProteinSimple, San Jose, CA, USA). The relative protein levels were calculated using Image J software (Institutes of Health, Bethesda, MA, USA, https://imagej.nih.gov/ij/download.html, accessed on 27 April 2023).

### 2.10. HFD-Induced Obese Mice

The C57BL/6J male mice (8 weeks, 16–18 g) were purchased from Samtako (Osan, Republic of Korea) and maintained in experimental circumstances for 1 week. Then, the mice were randomly divided into five groups (*n* = 6 per group); a normal diet (ND), an HFD, an HFD + WIR (100 mg/kg/day), an HFD + EtIR (100 mg/kg/day), and an HFD + EGCG (50 mg/kg/day). To induce obesity after dividing the five groups, 60% kcal HFD (Rodent diet D12492) (Research Diet, New Brunswick, NJ, USA) was fed to the mice except for the ND group for 10 weeks. Each group of mice was housed in the same cage. WIR, EtIR, EGCG, or distilled water was administered via oral gavage once a day for 10 weeks. Body weight and food intake were checked once a week. All procedures used in animal experiments were performed according to a protocol approved by the Animal Care and Use Committee of the Institutional Review Board of Wonkwang University (WKU18-45).

### 2.11. Serum Analysis

The mice fasted for 3 h to collect serum before sacrificing. The serum was centrifuged at 4000× *g* for 10 min. FFA, serum glucose, total cholesterol (TC), high-density lipoprotein (HDL)-cholesterol, low-density lipoprotein (LDL)-cholesterol, aspartate aminotransferase (AST), alanine aminotransferase (ALT), blood urea nitrogen (BUN), and creatinine were measured by the Seoul Medical Science Institute (Seoul Clinical Laboratories, Seoul, Republic of Korea).

### 2.12. Hematoxylin and Eosin (H&E) Staining 

The eWAT was collected after sacrificing the mice. The tissues were fixed in 10% neutral buffered formalin for at least 24 h and embedded in paraffin after dehydrating. The paraffin blocks were cut into 5 μm paraffin sections, and H&E was stained. Microscopic images were obtained using a microscope (Leica, Wetzlar, Germany). For an average size of adipocytes, 10 randomly selected areas in microscopic images of eWAT were measured using Image J software.

### 2.13. Chromatography Analysis of WIR and EtIR

The liquid chromatography-mass spectrometry (LC-MS) analysis was conducted by the Natural Product Institute of Science and Technology (Anseong, Republic of Korea). The LC-MS analysis of WIR and EtIR was performed by Thermo Scientific Accela-HPLC System with Model 600 Pump, Accela Autosampler, Accela 80 Hz PDA Detector (200 to 800 nm), and a Thermo Scientific LTQ Velos Mass Spectrometer System (Thermo Fisher Scientific, Waltham, MA, USA). The specific analysis conditions are indicated in Table 2.

### 2.14. Statistical Analysis

GraphPad software version 8 was used for statistical analysis. Statistical analysis was performed using parametric one-way ANOVA with post hoc Tukey honestly significant difference (HSD) test for comparing multiple groups with normal distribution. For groups without a normal distribution, a non-parametric Kruskal–Wallis one-way analysis followed by Dunn’s multiple comparison method was used. Parametric data are represented as mean ± SD. Non-parametric data are represented as boxplots showing medians and 25th and 75th percentiles. Whiskers showed Min and Max values. *p*-value ≤ 0.05 was considered a significant difference. Data were visualized by GraphPad Prism (GraphPad Software).

## 3. Results

### 3.1. WIR and EtIR Reduced Lipid Accumulation of 3T3-L1 Cells

**Figure 1:**
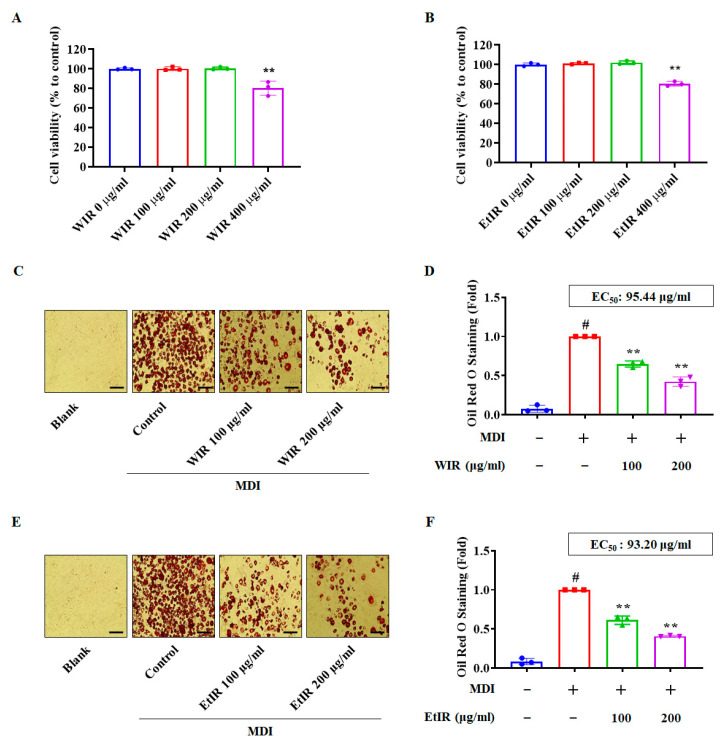
Effects of WIR and EtIR on lipid accumulation in 3T3-L1 cells. (**A**,**B**) The cell viability was calculated after treating indicated concentration of the WIR (**A**) or EtIR (**B**). Oil Red O staining images were captured after treatment with or without WIR (**C**). Lipid accumulation was measured by stained Oil Red O solution (**D**). Oil Red O staining images were captured after treatment with or without EtIR (**E**). Lipid accumulation was measured by stained Oil Red O solution (**F**). The magnification was 200×. Scale bar, 100 μm. All values are mean ± S.D. # *p* < 0.05, significantly different from MDI-untreated pre-adipocytes (Blank); ** *p* < 0.01 significantly different from MDI-treated adipocytes (control).

The 3T3-L1 cells were used to confirm the effect of WIR and EtIR. The murine pre-adipocyte 3T3-L1 cell has been widely used for investigating the molecular mechanism of lipid accumulation [27]. To determine the experimental concentration of WIR and EtIR, various concentrations of WIR and EtIR were treated in the cells. As shown in Figure 1A,B, treatment with WIR and EtIR up to 200 μg/mL did not induce cell cytotoxicity in 3T3-L1 cells. We used 200 μg/mL of WIR and EtIR as the highest concentration in our study. Next, we confirmed whether WIR and EtIR could reduce lipid accumulation in 3T3-L1 cells. Lipid droplets in 3T3-L1 cells were stained using an Oil Red O solution. In microscopic images, 100 and 200 μg/mL of WIR and EtIR treatment decreased lipid accumulation in a dose-dependent manner (Figure 1C,E). Then, the stained Oil Red O solution was extracted to compare the absorbance. As shown in Figure 1D,F, WIR and EtIR significantly decreased lipid accumulation, and EC_50_ of WIR and EtIR were 95.44 μg/mL and 93.20 μg/mL, respectively. This experiment confirmed that WIR and EtIR could reduce lipid accumulation.

### 3.2. WIR and EtIR Inhibited the Production of ROS through the CD36-AMPK Pathway during the Adipogenesis

**Figure 2:**
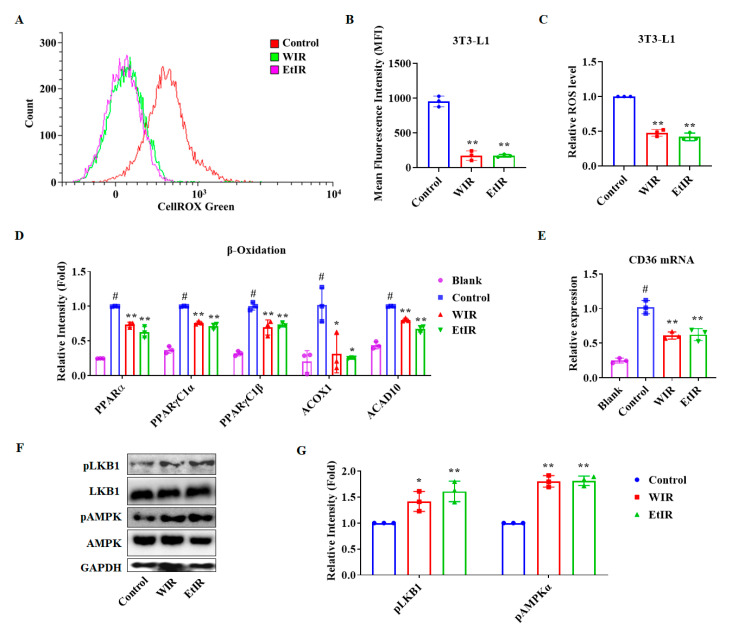
Effect of WIR and EtIR on the production of ROS, CD36-AMPK pathway, and β-oxidation during adipogenesis in 3T3-L1 cells. A total of 200 μg/mL of WIR and 200 μg/mL of EtIR were treated in the cells during the differentiation of 3T3-L1. (**A**,**B**) Cells were treated with CellROX for 30 min before analyzing to detect cellular oxidative stress levels. Confirmation of oxidative stress using flow cytometry (**A**). Mean fluorescence intensity (MFI) (**B**). Relative ROS level (**C**). mRNA levels of β-oxidation-related factors (*PPARα*, peroxisome proliferator-activated receptor alpha; *PPARγC1α*, peroxisome proliferator-activated receptor-gamma coactivator-1 alpha; *PPARγC1β*, peroxisome proliferator-activated receptor-gamma coactivator-1 beta; *ACOX1*, Acyl-CoA Oxidase 1; *ACAD10*, Acyl-CoA dehydrogenase family member 10) were measured in 3T3-L1 cells after WIR and EtIR treatment (**D**). The *CD36* mRNA level was analyzed by real-time RT-PCR (**E**). *GAPDH* was used as endogenous control. The effects of WIR and EtIR on the protein levels of pLKB1 and pAMPK were determined by western blot (**F**). The relative protein expression levels of pLKB1, LKB1, AMPK, and pAMPK were calculated by Image J software (**G**). GAPDH was used as endogenous control. All values are mean ± S.D. # *p* < 0.05, significantly different from MDI-untreated pre-adipocytes (blank); * p < 0.05, ** p < 0.01, significantly different from MDI-treated adipocytes (control).

Since ROS induced adipocyte differentiation [7], we confirmed the ROS amount after treating WIR and EtIR during adipocyte differentiation. First, we induced differentiation of 3T3-L1 and treated WIR or EtIR as indicated in materials and methods. As shown in Figure 2A–C, ROS production was significantly decreased by treatment of WIR and EtIR. In addition, increased β-oxidation-related genes by ROS were decreased by treatment of WIR and EtIR (Figure 2D). To confirm the mechanism between ROS and adipocyte differentiation, we checked the CD36-AMPK pathway. It has been reported that CD36 is essential for ROS generation [28,29]. Furthermore, depleted CD36 induced activation of AMPK; thus, regulation of CD36 is important for inhibiting lipid accumulation [28]. Compared to the control group, WIR and EtIR significantly decreased the expression level of *CD36* (Figure 2E). In addition, AMPK and its upstream protein, LKB1, were phosphorylated by treatment of WIR and EtIR (Figure 2F,G), indicating that WIR and EtIR inhibited ROS production by regulating the CD36-AMPK pathway.

### 3.3. WIR and EtIR Inhibited the Adipogenesis of 3T3-L1 Adipocytes

**Figure 3:**
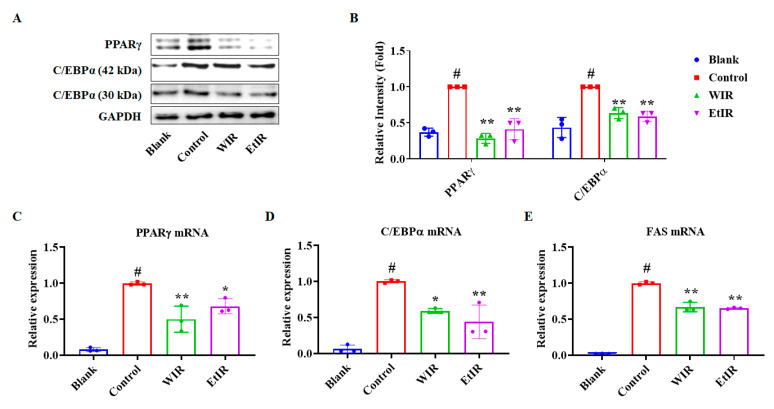
Effects of WIR and EtIR on adipogenesis in 3T3-L1 cells. A total of 200 μg/mL of WIR and 200 μg/mL of EtIR were treated in the cells during the differentiation of 3T3-L1, respectively. The effects of WIR and EtIR on the protein levels of PPARγ and C/EBPα were measured by western blot (**A**). The relative protein expression levels of PPARγ and C/EBPα were calculated by Image J software (**B**). (**C**–**E**) mRNA levels of *PPARγ* (**C**), *C/EBPα* (**D**), and *FAS* (**E**) were measured in WIR and EtIR-treated 3T3-L1 cells. GAPDH was used as endogenous control. All values are mean ± S.D. *# p* < 0.05, significantly different from MDI-untreated pre-adipocytes (blank); * *p* < 0.05, ** *p* < 0.01, significantly different from MDI-treated adipocytes (control).

Adipogenesis is the process by which pre-adipocytes differentiate into mature adipocytes. Adipogenesis is a multistep process that requires the activation of transcription factors, such as PPARγ and C/EBPα [30]. In addition, it was also reported that PPARγ and C/EBPα were regulated by CD36 [11]. Since WIR and EtIR reduced the expression of CD36 in our experiment, we confirmed whether WIR and EtIR reduced PPARγ and C/EBPα. As shown in Figure 3A–D, PPARγ and C/EBPα were down-regulated. Additionally, the mRNA expression level of *FAS* and the downstream gene of *PPARγ* and *C/EBPα* [31] were reduced by treatment of WIR and EtIR (Figure 3E).

### 3.4. WIR and EtIR Inhibited hAMSCs Differentiation into Adipocytes by Suppression of ROS

**Figure 4:**
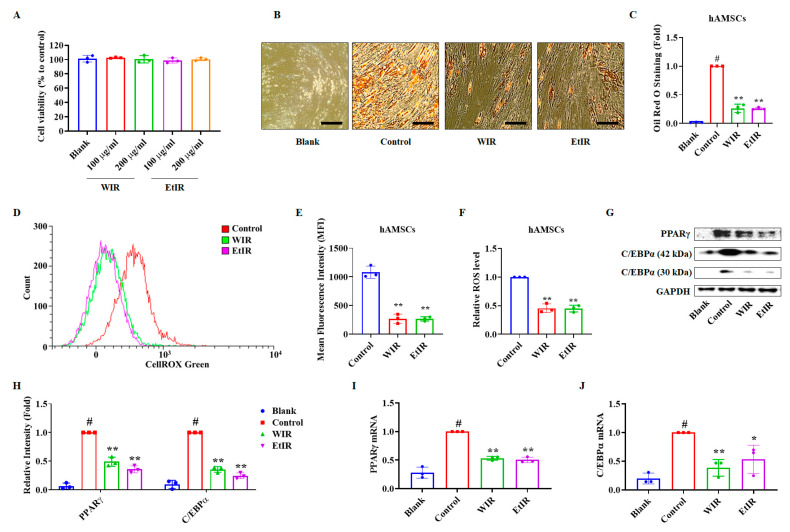
Effects of WIR and EtIR on differentiation of hAMSCs. The effects of WIR and EtIR on the cell viability were determined by MTS assay (**A**). Oil Red O staining images were captured after treatment with 200 μg/mL of WIR and 200 μg/mL of EtIR. The magnification was 200×. Scale bar, 50 μm (**B**). Relative lipid accumulation was measured by eluting stained Oil Red O solution from hAMSCs (**C**). (**D**,**E**) Differentiated hAMSCs were treated with CellROX for 30 min before analyzing to detect cellular oxidative stress levels. Confirmation of oxidative stress using flow cytometry (**D**). Mean fluorescence intensity (MFI) (**E**). Relative ROS level (**F**). (**G**,**H**) The protein levels of PPARγ and C/EBPα were determined by western blot. GAPDH was used as endogenous control (**G**). The relative levels of PPARγ and C/EBPα were calculated (**H**). (**I**,**J**) *PPARγ* (**I**) and *C/EBPα* (**J**) mRNA levels were measured by real-time RT-PCR. *h36b4* was used as endogenous control. All values are mean ± S.D. *# p* < 0.05, significantly different from DM-untreated hAMSCs (blank); * *p* < 0.05, ** *p* < 0.01, significantly different from DM-treated hAMSCs (control).

hAMSCs are isolated stem cells from adult human adipose tissue. Since they are able to differentiate into mature adipocytes, many studies have used hAMSCs to predict the clinical response [32]. We also differentiated hAMSCs into adipocytes to confirm the anti-adipogenic effect of WIR and EtIR. In order to select the experimental concentration of WIR and EtIR, hAMSCs were treated with 100 and 200 μg/mL of WIR and EtIR. As shown in Figure 4A, WIR and EtIR did not decrease cell viability. Next, we conducted Oil Red O staining to elucidate whether WIR and EtIR inhibited the differentiation of hAMSCs into adipocytes. Consistent with the results of 3T3-L1, treatment of 200 μg/mL of WIR and EtIR significantly reduced lipid accumulation compared to the control group (Figure 4B,C). In addition, WIR and EtIR reduced ROS production (Figure 4D–F). Protein levels and mRNA levels of PPARγ and C/EBPα were also significantly decreased by treatment of WIR and EtIR (Figure 4G–J). Therefore, this result strongly supported that WIR and EtIR inhibited adipogenesis by suppressing ROS production.

### 3.5. WIR and EtIR Reduced Body Weight and Regulated Serum Lipid Levels in Obese Mice

**Figure 5:**
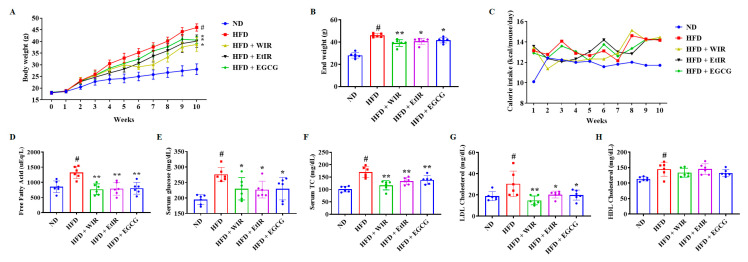
Effects of WIR and EtIR on weight gain and serum levels in HFD-induced obese mice. Weight changes for 10 weeks (**A**), the end weights of each group (**B**), and the amounts of food intake (kcal/mouse/day) (**C**) were measured. The levels of free fatty acid (**D**), glucose (**E**), TC (**F**), LDL cholesterol (**G**), and HDL cholesterol (**H**) were analyzed after sacrificing the mice. EGCG was used as a positive control drug for weight gain. ND, normal chow diet group; HFD, high-fat diet group; HFD + WIR, a high-fat diet plus WIR (100 mg/kg/day) group; HFD + EtIR, a high-fat diet plus EtIR (100 mg/kg/day) group; a high-fat diet plus EGCG (50 mg/kg/day) group (*n* = 6). All values are mean ± S.D. *# p* < 0.05, significantly different from the ND group; * *p* < 0.05, ** *p* < 0.01, significantly different from the HFD group.

The HFD-induced obese mice model was established to confirm whether the administration of WIR and EtIR could reduce weight gain. Mice were divided into five groups (*n* = 6 per group): ND, HFD, HFD with WIR (100 mg/kg/day), HFD with EtIR (100 mg/kg/day), and HFD with EGCG (50 mg/kg/day). In this experiment, EGCG, which is a bioactive compound of green tea, was used as a positive control drug for weight loss [13,16,33]. During the 10 weeks, the ND group gained 10.08 ± 1.64 g of weight, while the HFD group gained 28.06 ± 0.95 g. However, administration of WIR and EtIR decreased weight gain compared to the HFD group. The HFD + WIR group gained 20.73 ± 2.46 g, the HFD ± EtIR group gained 22.04 ± 2.35 g, and the HFD ± EGCG group gained 22.37 ± 1.96 g. The inhibitory effect of weight gain in the HFD + WIR group and HFD + EtIR group was similar to that of the HFD + EGCG group (HFD + WIR vs. HFD + EGCG; *p* = 0.3961, HFD + EtIR vs. HFD + EGCG; *p* = 0.9554) (Figure 5A,B). During this period, the daily calorie intake of the HFD + WIR group and HFD + EtIR group did not differ from the HFD groups (Figure 5C), suggesting that the effect of WIR and EtIR was not related to the reduction of calorie intake. Additionally, mice serum was analyzed after sacrificing the mice. It has been reported that HFD elevates serum free fatty acid (FFA), which could generate mitochondrial ROS [34,35]. As shown in Figure 5D, the administration of WIR and EtIR significantly reduces serum FFA. Furthermore, the levels of glucose, total cholesterol (TC), and LDL-cholesterol were decreased by administration of WIR, EtIR, and EGCG compared to the HFD group (Figure 5E–G). However, there were no significant differences in HDL-cholesterol (Figure 5H). During this period, treated drugs did not induce hepatic and renal toxicity (Table 3). These results suggested that WIR and EtIR could decrease weight gain without appetite change and drug toxicity.

### 3.6. WIR and EtIR Decreased Lipids in eWAT by Inhibiting Adipogenesis and β-Oxidation

**Figure 6:**
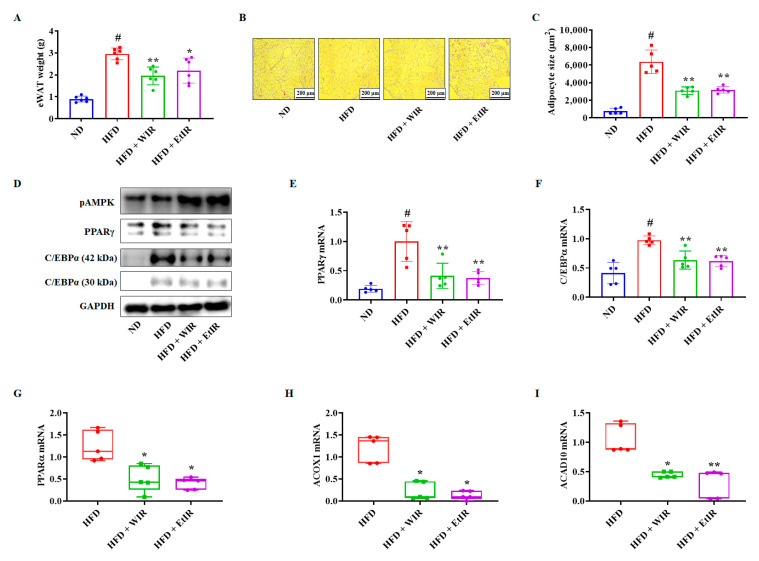
Effects of WIR and EtIR in eWAT from HFD-induced obese mice. The weights of eWAT (**A**). The H&E staining images of eWAT (**B**). The scale bar is 200 µm. The adipocytes of eWAT were randomly examined using Image J, and the size was quantified (**C**). The protein levels of pAMPK, PPARγ, and C/EBPα in eWAT were determined using a western blot. GAPDH was confirmed as endogenous control (**D**). (**E**–**I**) The mRNA levels of *PPARγ* (**E**), *C/EBPα* (**F**), *PPARα* (**G**), *ACOX1* (**H**), and *ACAD10* (**I**) were measured in eWAT. In panels (**A**,**C**,**E**,**F**) all values are mean ± S.D and analyzed by a one-way ANOVA. In panes (**G**–**I**), the boxes show the medians with the 25th and 75th percentiles, and the whiskers show the Min and Max values. The data were analyzed by a non-parametric Kruskal–Wallis one-way analysis followed by Dunn’s multiple comparison method. # *p* < 0.05, significantly different from ND group; * *p* < 0.05, ** *p* < 0.01, significantly different from HFD group.

The weight of eWAT and adipogenesis-related factors in eWAT were analyzed. As shown in Figure 6A, the administration of WIR and EtIR significantly reduced the weights of eWAT compared to the HFD group. H&E staining was also performed to confirm the size change of adipocytes in eWAT. The adipocyte size of the HFD group was significantly larger than the ND group. However, administration of WIR and EtIR inhibited adipocyte enlargement (Figure 6B). The average adipocytes size of the HFD group was significantly increased compared to the ND group (ND: 761.25 ± 330.61 μm^2^; HFD: 6387.297 ± 1340.17 μm^2^). However, administration of WIR and EtIR was reduced compared to the HFD group (WIR: 3097.55 ± 427.35 μm^2^; EtIR: 3199.66 ± 377.74 μm^2^, respectively (Figure 6C). We also confirmed expressions of PPARγ and C/EBPα in eWAT. As shown in Figure 6D,F, protein and mRNA levels of PPARγ and C/EBPα were significantly decreased by WIR and EtIR administration. On the other hand, WIR and EtIR administration increased the phosphorylation of AMPK. In addition, β-oxidation-related genes, including *PPARα*, *ACOX1*, and *ACAD10*, were decreased by the administration of WIR and EtIR in eWAT (Figure 6G–I).

### 3.7. Chromatography Analysis of WIR and EtIR

**Figure 7:**
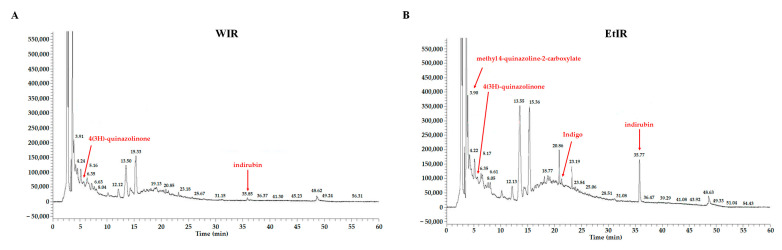
LC-MS-MS analysis of WIR and EtIR.

To validate WIR and EtIR, we analyzed the compounds composing WIR and EtIR through LC-MS-MS analysis. According to Deng et al., the contents of indigo and indirubin were one of higher components than that of other contained components in IR. In addition, results of the gastrointestinal absorption and drug-likeness analysis predicted that indigo and indirubin have the most physiological activity among the various components of IR [36]. As shown in Figure 7A, indirubin was detected in WIR. Additionally, indigo and indirubin were detected in EtIR (Figure 7B). These two compounds were especially extracted better by ethanol than water [37,38], the result which is consistent with our LC-MS-MS analysis (Figure 7). Therefore, we validated our IR extracts by detecting indigo and indirubin.

## 4. Discussion

Obesity is a major health problem worldwide. The typical phenotypes of obesity are adipocyte differentiation and intracellular lipid accumulation caused by metabolic imbalances between energy stored and consumed. In obese conditions, adipocytes increase their size, and enlarged adipose tissues induce dysfunctional and pro-inflammatory states by releasing FFA, ROS, and pro-inflammatory cytokines [39]. Due to these reasons, adipose tissue has been recognized as an endocrine organ rather than a simple energy storage tissue. The secreted factors from the adipose tissue play an important role in human physiology and pathology [40,41]. Various side effects including hepatotoxicity and nephrotoxicity [42], have been reported for several Food and Drug Administration (FDA) approved drugs, such as Orlistat Semaglutide and tirzepatide [43]. For these reasons, many studies tried to develop alternative anti-obesity drug [44,45,46].

The hallmark of obesity is weight gain derived from the excess lipid accumulation in the body [47]. In this study, 10 weeks of administration of WIR and EtIR prevented body weight gain induced by HFD. Since adult fat mass expansion was mainly induced by adipocyte hypertrophy [48,49], the suppression of eWAT weight increase from adipocyte hypertrophy is crucial for ameliorating obesity. Administration of WIR and EtIR successfully decreased the weight of eWAT and the size of adipocytes in eWAT. Lipid accumulation is closely related to hyperplasia as well as hypertrophy. A decreasing number of adipocytes is also important due to the lipid-storing capacity in the WAT. Adipogenesis, also known as adipocyte hyperplasia, is a differentiation process from pre-adipocytes to mature adipocytes and determines the number of adipocytes. Therefore, the regulation number of adipocytes, as well as the size of adipocytes, is a possible therapeutic approach for treating obesity [50]. PPARγ and C/EBPα are key transcription factors of adipogenesis and trigger adipogenesis [51]. WIR and EtIR significantly suppressed the expression of PPARγ and C/EBPα in eWAT. Thus, our HFD-induced obese mice experiment elucidated that the IR extract inhibited weight gain by reducing the enlargement of adipocytes and adipogenesis. Interestingly, the administration of WIR and EtIR significantly affected several factors in the serum related to obesity. The administration of WIR and EtIR especially lowered serum-fasting glucose and FFA, which could induce oxidative stress by increasing the production of ROS [52]. In addition, FFA increased by obesity contributes to dyslipidemia, characterized by high total cholesterol, high LDL cholesterol, and low HDL cholesterol [53,54]. In our results, the administration of WIR and EtIR decreased total cholesterol and LDL-cholesterol even though there was no effect on HDL-cholesterol.

Oxidative stress is caused by an imbalance between the production of ROS and antioxidant defenses in cells and tissues [55]. Oxidative stress induces chronic inflammation as well as a wide range of diseases, such as chronic obstructive pulmonary disease (COPD), atherosclerosis, Alzheimer’s disease, and cancer [56,57,58]. Epidemiological studies in humans and animals have reported that oxidative stress is closely associated with the pathogenesis of obesity and its related risk factors [59]. In addition, it has been reported that oxidative stress triggers obesity by inducing adipocyte differentiation and increasing the size of mature adipocytes [6]. ROS level was increased during the differentiation of 3T3-L1 pre-adipocytes into adipocytes, and several studies have already confirmed that ROS inhibition reduces lipid accumulation [8,60]. Although IR exerted an antioxidant effect in macrophages [24], there was no study on the antioxidant effect associated with adipocyte differentiation and the obese animal model; thus, we selected IR extract to confirm whether IR could be a potential anti-obesity drug by regulating ROS level. In our study, WIR and EtIR suppressed ROS production induced by adipocyte differentiation. In addition, β-oxidation, known as the source of increased ROS production [61], related genes were decreased by WIR and EtIR in in vitro and in vivo. Additionally, AMPK was activated by WIR and EtIR in the 3T3-L1 cells and eWAT. Since AMPK regulates adipocyte differentiation [62], many obesity studies have focused on AMPK regulation. It has been reported that the expression of CD36 suppressed activation of AMPK [28] and induced the formation of ROS [63]. The expression of CD36 was reduced by WIR and EtIR. As a result, AMPK was activated by WIR and EtIR, and activated AMPK inhibited adipogenesis by decreasing the expression of transcription factors, PPARγ and C/EBPα, which regulate adipogenesis [64]. Therefore, our results suggested that WIR and EtIR reduced lipid accumulation by regulating ROS production and adipogenesis-related pathway, indicating that IR extract could be a potential antioxidative drug by regulating oxidative stress-related factors.

Although we first elucidated the antioxidant property of IR in the obesity model, further research is needed to determine specific molecular mechanisms. Since we used an extract of a natural product, it is difficult to clarify which compound could suppress ROS before confirming the effect of a single compound in the current model. In this study, we only used a positive control to confirm mice weight loss due to the lack of an exact mechanism-matched drug in 3T3-L1 and hAMSCs. Although there was an exactly matched study on adipocytes, Moon et al. reported that metformin, widely studied on obesity, alleviated oxidative and ER stress induced by CD36 expression in pancreatic beta cells [65]. Therefore, metformin can serve as a potential positive control drug on oxidative stress and obesity, and consequently, its effect can be compared with metformin to prove the drug superiority of the natural product. Extraction methods are also important for exploring the effect of natural products. Since natural products were traditionally extracted using hot water in East Asia, various studies on natural products, including *Isatidis Radix*, still have employed a hot water extraction method to explore their effect. It is known that the hot water extract method could exhibit thermolabile properties on their bioactive compounds, despite the high solubility and diffusion [66]. In our result, amounts of indigo and indirubin were also more in ethanol extraction than in hot water extraction. Although we can fully explore the effect of IR in this study by extracting IR in two ways using hot water and ethanol to avoid degradation issues while still employing the traditional extraction method, the profiling study on extract amount for water temperature needs more support. Another limitation is whether administering WIR and EtIR could show concentration-dependent effects in animal experiments. According to the study by Nair et al. [67], 100 mg/kg/day for mice could be converted into 8 mg/kg/day for humans. Regarding our extraction yield, 2.67 g of IR is needed for WIR administration to 60 kg humans, and 1.77 g of IR is needed for EtIR administration to 60 kg humans. However, body systems chemically alter most drugs to excrete compounds more easily from the body [68]. It has been reported that indirubin, one of the major compounds of IR, could be metabolized into indigo carmine by CYP1A1, CYP1A2, or CYP1B1 in liver [69]. Therefore, although it is possible to calculate the administration concentration of IR in humans and no toxic effects were observed in mice, caution should be exercised when considering the use of IR in humans until further studies, such as on drug metabolism, are completed. We successfully proved that the current concentration of WIR and EtIR showed anti-obesity properties without any drug toxicity; further pharmacokinetics and pharmacodynamics studies are demanded before starting a clinical trial.

## 5. Conclusions

Taken together, this study demonstrated that WIR and EtIR suppressed lipid accumulation through the antioxidant mechanism in 3T3-L1 and hAMSCs. Moreover, administration of WIR and EtIR showed an inhibitory effect on weight gain in obese mice by regulating oxidative stress-related factors. Therefore, our study suggested that administering WIR and EtIR could be a therapeutic agent for treating obesity by regulating oxidative stress.

## Figures and Tables

**Table 1:** The primer sequences used for real-time RT-PCR.

Gene (Accession Number)	Sequences
*mPPARα* (XM_030248424)	5′-AACTGGATGACAGTGACATTTCC-3′ (sense)
	5′-CCCTCCTGCAACTTCTCAAT-3′ (antisense)
*mPPARγC1α* (XM_006503775)	5′-CCCTGCCATTGTTAAGAC-3′ (sense)
	5′-GCTGCTGTTCCTGTTTTC-3′ (antisense)
*mPPARγC1β* (XM_006525698)	5′-GAGGGCTCCGGCACTTCC-3′ (sense)
	5′-GTACTTGCTTTTCCCAGATG-3′ (antisense)
*mACOX1* (NM_001377522)	5′-GCATCGCAGACCCTGAAGAA-3′ (sense)
	5′-GGTGCATCCATTTCTCCTGC -3′ (antisense)
*mACAD10* (XM_030254818)	5′-CTTGGAGAAGTACCTGAAGG-3′ (sense)
	5′-CAGCCTGATGTAGTACGTTG-3′ (antisense)
*mCD36* (XM_030254088)	5′-ATGGGCTGTGATCGGAACTG-3′ (sense)
	5′-GTCTTCCCAATAAGCATGTCTCC-3′ (antisense)
*mPPARγ* (XM_017321455)	5′-TTTCAAGGGTGCCAGTTTC-3′ (sense)
	5′-TTATTCATCAGGGAGGCCAG-3′ (antisense)
*mC/EBPα* (NM_001287514)	5′-GCCGAGATAAAGCCAAACAA-3′ (sense)
	5′-CGTAAATGGGGATTTGGTCA-3′ (antisense)
*mFAS* (XM_030245556)	5′-TGGTGGGTTTGGTGAATTGTC-3′ (sense)
	5′-GCTTGTCCTGCTCTAACTGGAAGT-3′ (antisense)
*mGAPDH* (NM_001411843)	5′-AACTTTGGCATTGTGGAAGG-3′ (sense)
	5′-GGATGCAGGGATGATGTTCT-3′ (antisense)
hPPARγ (NM_001354666)	5′-TGAATGTGAAGCCCATTGAA-3′ (sense)
	5′-CTGCAGTAGCTGCACGTGTT-3′ (antisense)
hC/EBPα (NM_001287424)	5′-TGTATACCCCTGGTGGGAGA-3′ (sense)
	5′-TCATAACTCCGGTCCCTCTG-3′ (antisense)
h36b4 (NM_001002)	5′-AAACTGCTGCCTCATATCCGG-3′ (sense)
	5′-TTGTAGATGCTGCCATTGTCGA-3′ (antisense)

**Table 2:** LC-MS-MS conditions for analysis of WIR and EtIR.

Parameter	Condition	
Flow Rate	1.0 mL/min	
Column	YMC Pack-Pro C18	
Injection Vol.	20 μL	
Column Temp.	30 °C	
Heater Temp.	250 °C	
Sheath Gas Flow Rate	35 arb (N2)	
Spray Voltage	5 kV	
Capillary Temp.	275 °C	
Gradient condition		
Time (min)	Water (0.1% formic acid)	Acetonitrile (0.1% formic acid)
10	83	17
40	30	70
45	0	100
50	83	17
60	83	17

**Table 3:** Effects of WIR and EtIR on serum levels in HFD-induced obese mice.

	ND	HFD	HFD + WIR	HFD + EtIR
AST (IU/L)	142.67 ± 24.07	185.17 ± 51.70	159.83 ± 62.23	171.50 ± 52.08
ALT (IU/L)	41.33 ± 10.89	135.167 ± 67.72 ^#^	60.83 ± 38.25	123.67 ± 58.00
Creatinine (mg/dL)	0.16 ± 0.03	0.16 ± 0.03	0.15 ± 0.06	0.18 ± 0.04
BUN (mg/dL)	17.00 ± 1.26	22.33 ± 2.42 ^#^	14.17 ± 3.19 *	12.67 ± 2.16 *

ND, normal chow diet group; HFD, high-fat diet group; HFD + WIR, a high-fat diet plus WIR (100 mg/kg/day) group; HFD + EtIR, a high-fat diet plus EtIR (100 mg/kg/day) group (*n* = 6). All values are mean ± S.D. *^#^ p* < 0.05, significantly different from the ND group; * p < 0.05, significantly different from the HFD group.

## Data Availability

The data presented in this study are available on request from the corresponding authors.
